# In-person versus electronic screening for social risks among carers of pediatric inpatients: A mixed methods randomized trial

**DOI:** 10.1007/s00431-024-05470-1

**Published:** 2024-03-01

**Authors:** Lisa Gaye Smithers, Catherine MacPhail, Lily Chan, Maeve Downes, Kate Neadley, Mark Boyd

**Affiliations:** 1 School of Health and Society, University of Wollongong, Northfields Road, Wollongong, NSW 2522 Australia; 2 School of Public Health, University of Adelaide, Adelaide, SA Australia; 3 Northern Adelaide Local Health Network, Elizabeth Vale, SA Australia; 4 Adelaide Medical School, Faculty of Health and Medical Sciences, The University of Adelaide, Adelaide, SA Australia

**Keywords:** Pediatrics, Social determinants of health, Social disadvantage, Social risks, Screening modality, Mixed methods

## Abstract

We aimed to compare disclosure of social risks according to self-report on an iPad versus face-to-face questions from a health professional and to explore carers’ experiences of screening. This two-arm, parallel group, randomized trial was conducted from January 19, 2021, to December 17, 2021, in a public hospital pediatric ward serving a disadvantaged area of an Australian capital city. Carers of children aged ≤ 5 years admitted to the Children’s Ward were eligible. The primary outcome was disclosure of social risks. The screener included nine items on food security, household utilities, transport, employment, personal and neighborhood safety, social support, housing and homelessness. Disclosure of social risks was similar between the self-completion (*n* = 193) and assisted-completion (*n* = 193) groups for all 9 items, ranging 4.1% higher for worrying about money for food (95% CI − 11.4, 3.1%) among the assisted-completion group, to 5.7% (−1.6, 13.0%) higher for unemployment among the self-completion group. In qualitative interviews, participants were positive about screening for social risks in the hospital ward setting and the majority indicated a preference for self-completion.

*Conclusion*: Differences in the disclosure of social risks according to self- versus assisted-completion were small, suggesting that either method could be used. Most carers expressed a preference for self- completion, which is therefore recommended as the ideal mode for such data collection for Australian pediatric inpatient settings.

*Trial registration*: Australia New Zealand Clinical Trial Registry (www.anzctry.org.au; #ACTRN12620001326987; date of registration 8 December 2020).

**Table:** 

**What is Known:** *• Most evidence on screening of social risks in pediatric inpatient settings is from the USA.* *• Little is known about disclosure of social risks in countries with universal health care and social welfare.*	
**What is New:** *• Disclosure of social risks was similar for electronic compared with face-to-face screening.* *• Carers preferred electronic completion over face-to-face completion.*

**Supplementary Information:**

The online version contains supplementary material available at 10.1007/s00431-024-05470-1.

## Introduction

Integrated health and social care systems have been suggested to address the real-life impacts of the social determinants of health [1]. Issues such as housing, financial security, social supports, and exposure to violence (among others) are increasingly recognized as social risks, which may be identified and addressed in healthcare settings. In the UK, primary care providers are incentivized to undertake “social prescribing,” where patients’ social risks are identified during primary care and they are referred to a link worker and appropriate community services. England’s NHS Long Term Plan committed to building social prescribing infrastructure and aims for 900,000 people to be referred to a social prescribing link worker by 2024 [2]. Portugal, Germany, and other European nations are embedding social prescribing in their primary care systems [3]. If social prescribing is to “level up” the gradient in health [4], then screening and referral interventions should occur in hospital settings to overcome the barriers that disadvantaged patients may face to access primary care [5]. Even in wealthy countries with universal healthcare such as Australia, disadvantaged populations struggle to access primary care [6].

Previously, we pilot tested a social risks screening tool at an Australian hospital in a disadvantaged area, where 95% of adult patients experienced one or more adverse social determinants of health [7]. Despite working in areas of high disadvantage, some hospital clinicians do not routinely enquire about social risks [8] even though this may reduce stigma and improve patient management. Numerous professional organizations have recommended screening for social risks [9], with the American Academy of Pediatrics calling for universal screening in pediatric settings [10], as there are likely longer-term repercussions for children’s health and wellbeing. However, the optimal mode and acceptability of collecting social risk information are not clear. Apart from the notable iScreen trial [11], there are very few high quality randomized trials that have tested screening for social risks in pediatric inpatient settings, particularly in countries with universal healthcare. We address this gap in evidence by undertaking a randomized trial in an Australian hospital pediatric ward to test whether carers’ disclosure of social risks differed according to whether participants self-completed (via an iPad) compared with being asked by a health professional (assisted-completion). We also explored the acceptability of asking about social risks.

## Methods

This was a single-center, two-arm, parallel group, randomized trial. The Central Adelaide Local Health Network Human Research Ethics Committee (HREC #13717) approved the trial. Informed consent was obtained from individual participants. The trial was registered with the Australia New Zealand Clinical Trial Registry (#ACTRN12620001326987).

### Eligibility

Ward staff alerted researchers to admissions aged ≤ 5 years. Researchers then undertook eligibility screening. The researcher allowed carers’ to decide who would participate if more than one carer was available and volunteered. Inclusion criteria were adult (≥ 18 years old) parents or legal guardians of a child aged ≤ 5 years admitted to the Children’s Ward. Exclusion criteria were participating in the trial during previous admissions and inability to communicate in English.

### Setting

The study took place from 19 January 2021 to 17 December 2021 at the Northern Adelaide Local Health Network (NALHN), South Australia. The main NALHN hospital is in one of the most disadvantaged areas of any Australian city, with higher unemployment and housing stress than other metropolitan areas [12].

### Interventions

Participants assigned to the self-completion group were given an iPad to answer nine questions screening for social risks. The screening tool was based on a qualitative Australian study [13], reviewed in the context of the successful US-based Health Leads study [14], pilot tested in the same hospital as the present study [7], and then refined after consultation with senior Children's ward staff. Question topics were about housing security, homelessness, food security, household utilities, transport, personal safety, neighborhood safety, social support, and employment. Participants could decline to answer questions by skipping to the next question without providing a response. The questions and the order they were asked are in Supplementary Information (S1). Research staff left the room while participants completed the tool but remained nearby, if needed. The screener took < 10 min to complete. Participants assigned to the assisted-completion group had a research staff member ask the same questions, verbatim, and in the same order as presented to the self-completion group. Participants could respond to the researcher with yes or no, or decline to answer. Questions were asked immediately following randomization, with the whole in-person procedure of consenting, randomization, and responding to the questionnaire taking approximately 20 min. For both groups, all disclosures were reported to ward staff and responded to in line with current practice.

Participants in both groups were invited to a follow-up qualitative interview to give feedback on the experience. Participants were remunerated with a $5 voucher for the hospital cafeteria.

#### Protocol deviation

From 15 November 2021 to 26 November 2021, the admission procedure on the ward changed to incorporate the screening tool into routine practice. The research team met with ward staff, who agreed to temporarily revert to the previous admissions process (not using the screening tool) until the trial closed on 17 December 2021.

### Outcomes

The primary outcome is the difference in disclosure of social risks on each of the nine screening items for the self-completion versus assisted-completion groups. The difference in total count of social risks is a secondary outcome. Carers’ perceptions of the acceptability and feasibility of screening for social risks in the pediatric inpatient setting were the focus of qualitative interviews.

### Sample size

We used the US iScreen trial [11] and our previous research to guide the calculation [7]. iScreen reported 10% higher disclosure of sensitive questions (domestic violence, drug use) for self-completion than face-to-face completion. Thus, we aimed to enroll 200 participants per group (400 total) to detect a 10% difference in disclosure (10% versus 20% disclosure, 80% power, alpha 0.05).

### Randomization, allocation, and blinding

The trial statistician (LC) who was not involved in trial implementation prepared the randomization schedule using R software (1:1 allocation, no stratification, blocks drawn at random that ranged in size from 2 to 8). The randomization schedule remained hidden until each new enrollment. Trial staff confirmed eligibility and undertook informed consent and enrollment. After an enrollment, the REDCap database displayed the group allocation for new participants and the intervention was immediately implemented. It was not possible to blind the intervention, since the trial staff and participants knew whether the intervention was completed by themselves or with assistance.

### Qualitative interviews

Interviews were conducted within two weeks of the trial and followed a semi-structured schedule (Supplementary Information S2). Interviews were audio recorded, transcribed, and thematically analyzed using a social-constructivist perspective. Interviews took an average of 9.5 minutes. We regularly reviewed the demographic characteristics of interviewees to draw views from a range of gender (male, female), age (18–25 years versus > 25 years), disadvantage, and employment status. Interviewing ceased at 32 participants as no new insights were being obtained.

### Statistical analysis

Trial data was collected using REDCap software and analyzed using Stata/IC 15.1 (StataCorp LLC, College Station, TX, USA). Analyses proceeded according to a pre-written statistical analysis plan (available upon request).

Sociodemographic data is summarized descriptively. Socioeconomic position was obtained from residential postcode and converted to the Index of Relative Socioeconomic Advantage and Disadvantage (IRSAD) [15]. A “healthcare card holder” refers to an Australian government means-tested scheme that subsidizes healthcare for low-income families.

The primary endpoint was disclosure of each social need in the intention-to-treat (ITT) sample (i.e., according to group allocation), with the secondary endpoint being total number of social risks. A generalized binomial regression model with an identity link was used to estimate the mean difference in the prevalence of a social need in the self-completion group compared with assisted-completion. Homelessness and safety were analyzed by generalized normal regression (identity link, robust errors) due to convergence problems. For the secondary endpoint, total risks per participant (ranging 0–9) in the self-completion compared with assisted-completion were analyzed using a negative binomial model with robust errors due to overdispersion. Following peer-review, unplanned exploratory analyses included adjustments for age, sex, and education of participant (Supplementary Material). No participants were excluded from the ITT analyses. No adjustments were made for multiple analyses.

We anticipated that participants may decline to answer sensitive questions and a priori set a 5% threshold for addressing non-response in the analyses. The 5% threshold was not met; therefore, non-response analyses were not undertaken.

While the trial was underway, the screening tool was introduced for routine ward use, which meant that carers may have completed the screening tool prior to trial participation. A modified analysis was conducted in which 17 participants enrolled during these dates were excluded.

### Qualitative analysis

Interviews were conducted and transcribed by KN and imported into NVivo Version 12 for analysis by CM. We used a framework analytical approach that progressed through key phases [16]. These included data immersion by rereading transcripts multiple times, deductive generation of a code frame based on the interview topics and extant literature, and coding of data using the established code frame, but also allowing for inductive development of further codes driven by new ideas identified in the transcripts. Thereafter, we worked with the codes to define and review themes and sub-themes [17]. Trustworthiness of the analysis was supported by ensuring inductive thematic and a priori thematic saturation [18], interpretation of the transcripts, and reflexivity. Coding was conducted by CM and themes regularly discussed with coauthors.

## Results

**Fig. 1:**
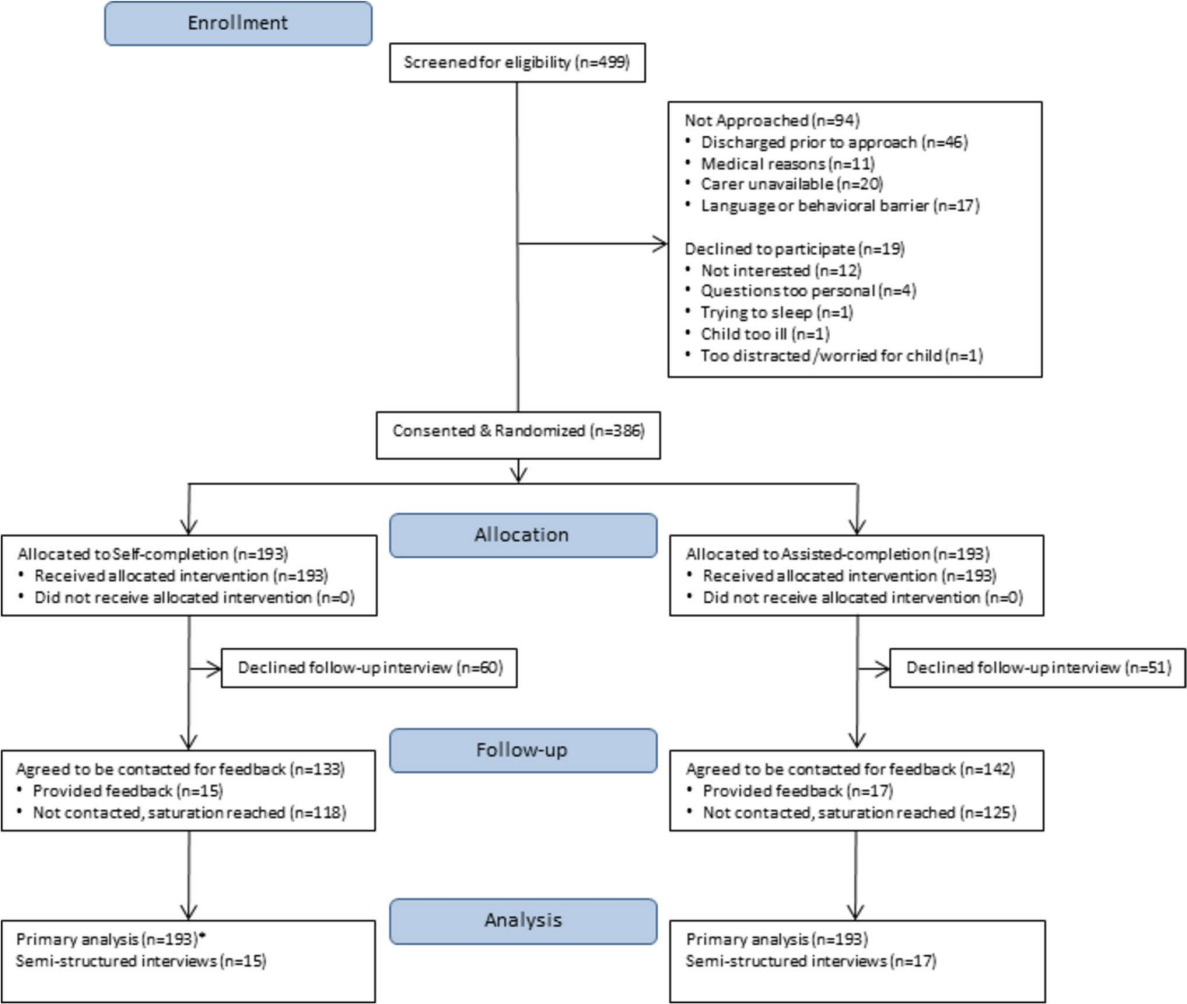
Flow of participants through the trial. *Missing data: item 2 of the screening tool (re: homeless/living in a shelter), *n* = 1; item 8 of the screening tool (re: support from family/friends/community services), *n* = 2; total number of social risks, *n* = 3

Of 499 people screened for eligibility, 193 were randomized to each group (Fig. 1). Table 1 shows sociodemographic characteristics were similar for the self-completion and assisted-completion groups.

**Table 1:** Sociodemographic characteristics of participants^a^

	Self-completion (*n* = 193)	Assisted-completion (*n* = 193)
Information about carer’s who completed the questionnaire		
Age, years (mean, SD)	30.3 (6.0)	30.8 (5.5)
Gender, No. (%)		
Male	31 (16.1)	27 (14.0)
Female	162 (83.9)	166 (86.0)
Place of birth, No. (%)		
Australia	156 (80.8)	162 (83.9)
Asia	20 (10.4)	20 (10.4)
Other countries	17 (8.8)	11 (5.7)
IRSAD quintile, No. (%)^a^		
Quintile 1 (most disadvantaged)	119 (61.7)	127 (65.8)
Quintile 2	26 (13.5)	23 (11.9)
Quintile 3	28 (14.5)	26 (13.5)
Quintile 4	19 (9.8)	15 (7.8)
Quintile 5 (most advantaged)	0 (0.0)	2 (1.0)
Not reported	1 (0.5)	0 (0.0)
Highest level of school completed, No. (%)		
Year 10 or lower	24 (12.4)	42 (21.8)
Year 11	40 (20.7)	41 (21.2)
Year 12	129 (66.8)	110 (57.0)
Enrollment in high education, No. (%)		
Yes	137 (71.0)	151 (78.2)
No	54 (28.0)	42 (21.8)
Not reported	2 (1.0)	0 (0.0)
Health care card holder, No. (%)		
Yes	94 (48.7)	89 (46.1)
No	99 (51.3)	104 (53.9)
Information about hospitalized child, No. (%)		
Male	104 (53.9)	103 (53.4)
Female	89 (46.1)	90 (46.6)

*IRSAD* Index of Relative Socioeconomic Advantage and Disadvantage

^a^Some percentages do not add up to 100% due to rounding

### Disclosure of social risks

Table 2 shows disclosure of social risks by each item. Of the nine questions asked of 396 participants (3474 items), only three were not answered (0.1%); two on social support and one on homelessness. Social risks was highest for worries about money for rent/mortgage (self-completion 18.1%; assisted-completion 20.2%), food (self-completion 13.5%; assisted-completion 17.6%) and not having paid work (self-completion 18.7%; assisted-completion 13.0%), and lowest for homelessness (self-completion 2.6%; assisted-completion 0.5%). The mean differences in disclosure of social risks ranged from 4.1% lower (95% CI − 11.4%, 3.1%) through to 5.7% higher (−1.6%, 13.0%) in the self-completion group compared with assisted-completion. The results were similar for the modified analysis, which excluded participants recruited during the period of changed admission practices (Table 2). The total number of social risks disclosed was 9.8% higher (95% CI − 17.2%, 45.7%; *p* = 0.516) among the self-completion group compared with assisted-completion, which was similar to the modified analysis (6.4% higher (95% CI − 20.2%, 41.9%); *p* = 0.672). Exploratory analyses with adjustments were similar to these findings (Supplementary Information S3), although the total number of social risks was 18.4% higher (95% CI − 11.2, 57.9; *p* = 0.249) among self-completion versus assisted completion.

**Table 2:** Differences in the disclosure of social risks according to self-completion versus assisted-completion

	Self-completion (*n* = 193)			Assisted-completion (*n* = 193)			ITT analysis	Modified analysis
	Yes*	No*	NR*	Yes*	No*	NR*	MD (95% CI), *p*	MD (95% CI), *p*
In the past 6 months were you worried that you did not have enough money to pay your rent and mortgage?	35 (18.1)	158 (81.9)	0 (0.0)	39 (20.2)	154 (79.8)	0 (0.0)	−2.1% (−9.9%, 5.8%), 0.605	−2.1% (−10.3%, 6.1%), 0.621
At any time in the last 6 months, were you and your family homeless or living in a shelter?	5 (2.6)	187 (96.9)	1 (0.5)	1 (0.5)	192 (99.5)	0 (0.0)	2.1% (−0.4%, 4.6%), 0.098	2.2% (−0.4%, 4.8%), 0.097
In the past 6 months were you worried that you did not have enough money for food for your family?	26 (13.5)	167 (86.5)	0 (0.0)	34 (17.6)	159 (82.4)	0 (0.0)	−4.1% (−11.4%, 3.1%), 0.260	−4.3% (−11.8%, 3.3%), 0.268
In the past 6 months were you unable to pay your electricity, gas or water bills?	35 (18.1)	158 (81.9)	0 (0.0)	28 (14.5)	165 (85.5)	0 (0.0)	3.6% (−3.7%, 11.0%), 0.334	2.8% (−4.8%, 10.4%), 0.469
In the past 6 months have you been unable to do your day to day activities, such as shopping, going to appointments, or work because you did not have transport?	18 (9.3)	175 (90.7)	0 (0.0)	11 (5.7)	182 (94.3)	0 (0.0)	3.6% (−1.6%, 8.9%), 0.175	3.3% (−2.1%, 8.7%), 0.232
In the past 6 months did you feel that you or your family were not safe in your home environment?	15 (7.8)	178 (92.2)	0 (0.0)	8 (4.1)	185 (95.9)	0 (0.0)	3.6% (−1.1%, 8.3%), 0.131	2.8% (−2.0%, 7.5%), 0.255
In the past 6 months did you feel that you or your family were not safe in your neighborhood?	26 (13.5)	167 (86.5)	0 (0.0)	29 (15.0)	164 (85.0)	0 (0.0)	−1.6% (−8.5%, 5.4%), 0.662	−2.6% (−9.8%, 4.5%), 0.470
In the past 6 months did you feel that you had support from family, friends or community services?^a^	170 (88.1)	21 (10.9)	2 (1.0)	171 (88.6)	22 (11.4)	0 (0.0)	−0.4% (−6.7%, 5.9%), 0.900	−0.4% (−6.8%, 6.1%), 0.912
Did you or anyone in your household undertake paid work in the last 6 months?^a^	157 (81.3)	36 (18.7)	0 (0.0)	168 (87.0)	25 (13.0)	0 (0.0)	5.7% (−1.6%, 13.0%), 0.124	5.5% (−1.9%, 13.0%), 0.145

*CI* Confidence interval, *IIT* Intention to treat analysis, *MD* Mean difference, *NR* Not reported

^a^This question was reverse coded for analysis, to indicate a lack of support/unemployment

*Column data showing *n*(%) and marking the Self-completion (*n *= 193) and Assisted-completion (*n *= 193) columns with the asterisk

### Semi-structured interviews

Interviews were conducted with 23 (72%) females and 9 (28%) males; mothers were more likely than fathers to accompany their children to the hospital and to agree to the interview. Parents were mostly aged > 25 years (81%) and employed (81%), although 50% of interviewees were from the most disadvantaged socioeconomic quintile. Representative quotations from interviews are included in Supplementary Information Table S3.

#### Ease of completion

Overall, there were strong positive attitudes towards the use of a screening tool to assist with referrals to services outside of the hospital. Interviewed participants agreed that items were easy to answer, most commenting they found it “straightforward” and estimated taking under 10 minutes.

#### Level of comfort and potential barriers

The majority of participants were comfortable answering the questions no matter which arm they had been allocated to but suggested that some families may be reluctant to answer potentially sensitive questions in assisted-completion delivery. No participants refused to answer specific questions, and when asked about refusals and sensitivity, none commented that they had found any questions too sensitive. A common theme however was reflecting that their own current situation did not warrant concerns about sensitivity, but individuals who were struggling could experience this differently. Sensitive questions were deemed to be about issues such as domestic violence, poverty and (un)employment. One participant who had previously had a violent and drug-addicted partner specifically reflected on this (quote in Supplementary Table 3). One of the Aboriginal participants in the study noted that life experience and intergenerational trauma of other Aboriginal patients might be a barrier to using a social screening tool.

Some participants felt “others” might appreciate assistance, particularly if they had low levels of literacy, but even participants who had initially been skeptical of their ability to self-administer the screen commented that they had managed without difficulty. In general, participants felt that most sensitivities could be overcome if the screening was adequately explained and patients understood the potential benefits of disclosing social issues for referrals. One of the participants reflected on a time when they had previously needed a referral and remarked on how useful it was to be offered assistance.

#### Preferred mode

Given that the trial specifically tested assisted-completion versus self-completion, we asked participants to reflect on their preferred mode. Participants were most in favor of self-completion (21 vs 5 responses from 32 interviews), arguing that it allowed for greater privacy, faster completion and potentially less embarrassment over sensitive information.

#### Screening in hospital

In terms of using the hospital as the location in which to conduct the screening, most participants felt that it was appropriate. When asked, participants struggled to think about other possible locations at which the screening would be useful but did mention GP practices, schools and community centers.

#### Question salience

Participants tempered many of their responses to the interview questions with comments about how they felt that the social issues in the screening tool were not currently of particular concern to them and that this influenced their responses to questions. However, when pressed to think about the social issues currently impacting their lives, many participants mentioned challenges with money, unemployment, housing, children, and domestic violence. In contrast, neighborhood safety was by far issue of least concern.

## Discussion

The current findings show that for carers of hospitalized children, disclosure of social risks is similar for those who self-completed questions using an iPad compared with being asked questions by a health-trained researcher. Additionally, the total number of social risks disclosed was similar across both groups. With only three of 3474 items (0.01%) declined to answer, the high (99.9%) response to all items supports the acceptability of the tool used regardless of mode of delivery. This was confirmed in the qualitative analysis, in which carers viewed the tool as acceptable, easy to use, and suitable for implementation in the pediatric inpatient setting. The qualitative analysis revealed a preference among carers for completion by self-administration.

With respect to comparing these findings with current evidence, our trial may be unique in Australia. Our findings are reasonably consistent with the US-based iScreen trial [11], the largest trial of mode of screening for social risks, although there are some differences. iScreen was implemented in an emergency department setting and involved a different tool with 23 questions. Although our tool was developed for the Australian context [7], both trials included items about housing, finance, food security, transport, employment, neighborhood, and domestic safety. Both iScreen and the current trial suggest that differences in disclosure due to mode of collection are minor for most domains. The exception may be domestic violence where iScreen reported 6.3% higher disclosure among self-completion compared with assisted completion (13.8% versus 7.5%). In our trial, disclosure was also higher among self-completed (7.8%) compared with assisted-completion (4.1%), but we observed lower rates of domestic violence and were underpowered to detect such a difference. To power a trial to detect 4% difference in disclosure would require > 1200 participants (alpha 5%, power 80%). Similarly, our qualitative findings about the acceptability of screening are consistent with other US-based studies involving screening [19, 20]. It is difficult to compare the current findings with European and UK contexts, as those studies commonly occur in primary care where populations, screening and referral workflows differ to inpatient settings. Testing the mode of screening in an inpatient setting in a country with universal healthcare sets this study apart from other literature.

Numerous systematic reviews have lamented the poor development of many social risks screening tools and called for better testing of such tools in randomized trials [21–23]. Although our tool was developed to meet local risks [7, 13], the domains are consistent with those described in a review of screening for social risks [24] and are considered some of the most important social determinants of health. Additionally, we attempted to improve internal validity by applying as many rigorous design elements as possible to limit potential biases. These included aspects of experimental design (e.g., individually randomized, independent statistician generating the randomization schedule that remained concealed until assignment) and best-practice implementation such as trial registration, pre-written protocol, and analysis plans. However, we acknowledge that participants and staff were not blinded to the mode of completion. The preference for self-completion might suggest some underreporting of social risks in the assisted-completion group, although the high completion rate, lack of withdrawals, and willingness to participate in qualitative interviews add confidence to these findings. Ultimately, it is not possible to know the extent to which the inability to blind the intervention affected the accuracy of outcome reporting and whether this differed by group allocation.

The introduction of the screening tool to routine practice had little effect on the findings but illustrated the deliberately pragmatic design and ease of translation to practice. Prior to commencing the trial, a social worker was stationed on the ward for an hour per day. Children’s Ward staff had identified integration of social determinants as critical to family functioning and supporting the admitted child’s health, and nursing staff were eager to assist families navigate pathways to support. Further work is needed to assess whether screening is acceptable among culturally and linguistically diverse communities, or in areas with different patterns of social risks.

Disclosure of social risks were similar between self- versus assisted completion groups. The current trial demonstrates acceptability of social risks screening in an Australian pediatric inpatient setting, of a highly disadvantaged area. Carers expressed a preference for self-completion, which is therefore recommend as the ideal mode for such data collection in Australian pediatric inpatient settings.

### Supplementary Information

Below is the link to the electronic supplementary material.

Supplementary file1 (DOCX 22 KB)

## Data Availability

The authors will share the trial protocol and data analysis plan upon request. Access to these documents can be made by emailing the Author for Correspondence (lsmithers@uow.edu.au). Deidentified individual participant data will not be made available as the authors do not have ethical approval or consent to share the trial data.

## Declarations

### Disclaimer

The funders had no role in the design and conduct of the study; collection, management, analysis, and interpretation of the date; preparation, review, or approval of the manuscript; and decision to submit the manuscript for publication.
