# Supplementary material for: In-person versus electronic screening for social risks among carers of pediatric inpatients: A mixed methods randomized trial
Source: Eur J Pediatr. 2024 Mar 1;183(5):2301–9. doi: 10.1007/s00431-024-05470-1 (PMC11035429; doi:10.1007/s00431-024-05470-1)
Supplement: Supplementary file 1 — Supplementary file1 (DOCX 22 KB) [file 431_2024_5470_MOESM1_ESM.docx]

**EUROPEAN JOURNAL OF PEDIATRICS - SUPPLEMENTARY INFORMATION**

**Screening for Social Needs among Carers of Pediatric Inpatients: A Mixed Methods Randomized Trial**

Lisa Gaye Smithers PhD^a,b^, Catherine MacPhail PhD^a^, Lily Chan PhD^b^, Maeve Downes GradDip (Health Management)^c^, Kate Neadley BSc(Hons)^d^, Mark Boyd MD^d^

**Affililations**

^a^ School of Health and Society, University of Wollongong, Wollongong NSW

^b^ School of Public Health, University of Adelaide, Adelaide, SA

^c^ Northern Adelaide Local Health Network, Elizabeth Vale SA

^d^ Adelaide Medical School, Faculty of Health and Medical Sciences, The University of Adelaide, Adelaide SA

**Author for correspondence**: Prof Lisa Smithers [lsmithers@uow.edu.au](mailto:lsmithers@uow.edu.au)

**Supplementary Information Table S1: Screening questions**

|  |  |  |
| --- | --- | --- |
| **HOUSING**: In the past 6 months were you worried that you did not have enough money to pay your rent and mortgage? | YES | NO |
| At any time in the last 6 months, were you and your family homeless or living in a shelter? | YES | NO |
| **FOOD:** In the past 6 months were you worried that you did not have enough money for food for your family? | YES | NO |
| **HOUSEHOLD BILLS:** In the past 6 months were you unable to pay your electricity, gas or water bills? | YES | NO |
| **TRANSPORT:** In the past 6 months have you been unable to do your day to day activities, such as shopping, going to appointments, or work because you did not have transport? | YES | NO |
| **SAFETY:** In the past 6 months did you feel that you and your family were not safe in your home environment? | YES | NO |
| In the past 6 months did you feel that you and your family were not safe in your neighborhood? | YES | NO |
| **SUPPORT:** In the past 6 months did you feel that you had support from family, friends or community services? | YES | NO |
| **EMPLOYMENT:** Did you or anyone in your household undertake paid work in the last 6 months? | YES | NO |

**Supplementary Information 2: Semi-structured interview schedule**

1) Please tell me about your experiences of filling out the questionnaire?

- Prompt:
  - i. How easy was it to complete?
  - ii. How long did it take?
  - iii. Were there any questions you felt you didn’t want to answer? (Housing, food, household bills, transport, safety, support, employment, demographics) and why?
  - iv. Would you prefer to complete it yourself or would you like help with it? Why? Who would you like to help you?

2) Some of the questions we asked in the questionnaire may have been quite sensitive, how did you feel about answering them?

- Prompt: Which of the questions did you think were sensitive?

3) If this questionnaire was used in the hospital in the future:

- Is the hospital a suitable place to ask these questions or would you have another suggestion?
- How useful do you think it is for parents/carers to be asked these questions while visiting this ward?
- If you identified an area of need from the questionnaire would you be interested in being referred by ward staff for assistance for that need?

4) Is there anything further you would like to comment on about the questionnaire or the process?

**Supplementary Information 3**

**Table S2: Unplanned exploratory analyses involving adjustments for age, sex and education^a^**

| **INDIVIDUAL ITEMS** | ITT analysis  MD (95% CI), p | Modified analysis  MD (95% CI), p |
| --- | --- | --- |
| In the past 6 months were you worried that you did not have enough money to pay your rent and mortgage? | -1.2% (-8.9%, 6.4%), 0.751 | -1.3% (-9.3%, 6.8%), 0.760 |
| At any time in the last 6 months, were you and your family homeless or living in a shelter? | 2.2% (-0.3%, 4.8%), 0.088 | 2.4% (-0.4%, 5.1%), 0.091 |
| In the past 6 months were you worried that you did not have enough money for food for your family? | -4.0% (-11.1%, 3.2%), 0.276 | -4.1% (-11.6%, 3.4%), 0.285 |
| In the past 6 months were you unable to pay your electricity, gas or water bills? | 5.0% (-1.9%, 11.9%), 0.154 | 4.0% (-3.0%, 11.1%), 0.261 |
| In the past 6 months have you been unable to do your day to day activities, such as shopping, going to appointments, or work because you did not have transport? | 2.4% (-2.6%, 7.4%), 0.344 | 1.6% (-3.5%, 6.8%), 0.536 |
| In the past 6 months did you feel that you or your family were not safe in your home environment? | 4.1% (-0.6%, 8.9%), 0.088 | 1.8% (-3.7%, 7.4%), 0.514 |
| In the past 6 months did you feel that you or your family were not safe in your neighbourhood? | -1.4% (-8.9%, 6.2%), 0.719 | -2.3% (-9.8%, 5.2%), 0.544 |
| In the past 6 months did you feel that you had support from family, friends or community services?^b^ | 0.7% (-5.7%, 7.1%), 0.833 | 0.8%. (-5.8%, 7.4%), 0.813 |
| Did you or anyone in your household undertake paid work in the last 6 months?^b^ | 7.7% (1.1%, 14.3%), 0.022 | 8.7% (1.7%, 15.8%), 0.015 |
|  | IRR (95% CI), p | IRR (95% CI), p |
| **TOTAL COUNT OF SOCIAL NEEDS**^b^ | 1.184 (0.888, 1.579), 0.249 | 1.139 (0.848, 1.530), 0.386 |

^a^ Assisted-completion group as reference

^b^ This question was reverse coded for analysis, to indicate a lack of support/unemployment

^c^ Abbreviations: CI, confidence interval; IRR, Incidence rate ratio; ITT, intention to treat analysis; MD, mean difference.

**Supplementary Information 4**

**Table S3: Quotations from qualitative analysis of screening for social needs**

| Concept | Participant information | Representative quotation/s |
| --- | --- | --- |
| Ease of completion | Older employed female, IRSAD 4^a^ | I think it was easy enough. I feel, like in the current situation where I was a bit sleep-deprived and you do have a lot of people coming in… you know, someone asking me if I want to use my private health [insurance], and the lunch lady came in, and I didn’t fill out my form, and nurses… I probably was a little bit more… found it more difficult than other circumstances. But it wasn’t overly difficult. |
| Level of comfort answering questions | Younger employed female, IRSAD 1  Older employed female, IRSAD 1  Older unemployed female, IRSAD 5 | I found the questions were pretty straightforward, but someone that was probably in a lot of trouble or not coping, probably wouldn’t want someone else filling it out or to do it full stop, because it compromises how they’re doing in coping in life.  The questions were clear and concise. I guess also found it easy from an emotional sense, because I’m not someone who has disadvantage. I think it perhaps could be more confronting for someone who is in a situation of disadvantage. Just for example, there’s no issues with family violence, but I guess, you know, when answering the questionnaire, if there was one person in the room and the other person was a perpetrator of family violence, it could be really difficult for that other person to answer.  I felt really good about answering them [the screening questions] because I am where I am. But if, yeah, that had been over a year ago, I probably would have been scared and . . . um . . . just not completely honest because I would like, like I said for the previous question . . . my partner. Yeah. |
| Potential barrier | Older employed female, IRSAD 3 | I’m . . . personally I’m fine. I guess . . . um . . . I just wanted to flag though that some people actually might be quite anxious in relation to answering that because, obviously, you know . . . um . . . Aboriginal people in particular are quite fearful of, you know, Child Protection or authorities, that they might be quite anxious. Like, “what does this mean if I am honest?” |
| Preferred mode of completion | Older employed female IRSAD 4  Older unemployed male, IRSAD 2  Older unemployed female, IRSAD 2 | I think one, just because I'm able, I'm quite comfortable using a device and I can read English. And I guess it just definitely gives it that more private feeling.  At the start I was a bit oohhh, like when you handed me the iPad. But you fill it out yourself, it's quite easy, but, you know, after I first started, you know as well because I'm not a person that uses, like, iPads or… I just use my mobile phone and it took me forever to learn how to do that, even send an email.  When I was . . . and went into the hospital and there was the offer for help, I took it right away because it wasn’t just about me, it was about her [child]. Me, as a parent, you might ask . . . I mean, you might worry that you’re gonna get judged, or obviously a lot of people might not understand… I think sometimes parents forget about themselves in the moment. |
| Screening in hospital setting | Older employed male, IRSAD 3 | I think that it is an excellent idea and I think it’s [the hospital] a good, safe environment for that to happen. |
| Question salience to self and community | Older employed male, IRSAD 3  Young employed female, IRSAD 1 | Um not a lot with us [issues of concern]. Yeah, we’re not comfortable. Well you know, comfortable. Everybody’s got bills, but you know, we’re pretty good.  The least worrying for me would be safety, I don’t have any problems . . . I would say, you know, we have transport issues all the time with cars and we have, you know, mortgages and stuff that bounces every now and then. So, yes, it’d be safety. We’re in a pretty safe area. That’s one less concern I would say. |

^a^ Abbreviation; IRSAD, Index of Relative Socioeconomic Advantage and Disadvantage
